# Identifying fundamental gaps in functional metagenomics: a step towards unlocking microbiome research potential

**DOI:** 10.1093/nargab/lqag110

**Published:** 2026-09-08

**Authors:** Sumeet K Tiwari, Andrea Telatin, Dipali Singh

**Affiliations:** Quadram Institute Bioscience, Norwich Research Park, Norwich NR4 7UQ, United Kingdom; Centre for Microbial Interactions, Norwich Research Park, Norwich NR4 7UG, United Kingdom; Quadram Institute Bioscience, Norwich Research Park, Norwich NR4 7UQ, United Kingdom; Centre for Microbial Interactions, Norwich Research Park, Norwich NR4 7UG, United Kingdom; Quadram Institute Bioscience, Norwich Research Park, Norwich NR4 7UQ, United Kingdom; Centre for Microbial Interactions, Norwich Research Park, Norwich NR4 7UG, United Kingdom

## Abstract

Incomplete functional annotation limits biological interpretation in microbiome studies and their translational potential. Poor annotation arises from multiple causes, with incomplete gene–protein–reaction mapping being one tractable yet under-examined contributor. We address this gap by developing a comprehensive hierarchical framework that systematically integrates gene families in UniRef, proteins in UniProt, and metabolic reactions in MetaCyc and BioCyc through UniProtKB accession, EC number, and Pfam-domain matching. Applied to a human gut metagenome dataset via HUMAnN3, our MetaCyc-based mapping recovers up to 2.3-fold more unique reaction identifiers than the default pipeline and increases reaction prevalence across samples from *≈*32% to 52% core reactions, addressing the data sparsity that limits statistical and machine-learning applications in microbiome research. Biological plausibility for the tested functions was supported by positive and negative controls: gut-microbial hormone-metabolism reactions previously linked to this dataset were recovered, while vertebrate-specific hormone-metabolism reactions remained correctly undetected. These gains derive from systematic database integration alone, without predictive algorithms, indicating that a tractable, mapping-related component of functional dark matter and data sparsity in microbiome studies is directly addressable. Because Pfam- and BioCyc-derived mappings trade specificity for coverage, confidence in any individual reaction assignment depends on the supporting evidence tier and source database.

## Introduction

The study of microbial communities has traditionally relied on taxonomic profiling to determine who is present, using phylogenetic markers or genome sequencing [1]. However, understanding microbial contributions to host physiology, disease, or ecosystem processes requires insight into what they do: the gene products, biochemical activities, and metabolic capabilities that mediate interactions with their environment or host [2]. This functional perspective is foundational to translating microbiome research into clinical diagnostics, therapeutic interventions, and biotechnological applications [3, 4]. Modern metagenomic studies increasingly recognize that taxonomic and functional perspectives are complementary. While taxonomy defines community structure and biogeographical patterns [5], functional profiling reveals metabolic potential, biochemical activity, and ecosystem-level functions [6, 7]. Integrating these perspectives links taxa to specific functions, identifies functional redundancy, and provides a mechanistic basis for assessing community metabolic capacity, which is a critical requirement for personalized medicine approaches that leverage microbiome modulation [8, 9].

Microbiome studies have widely converged on metabolic pathways as the primary metric of functional profiling [6, 10]. While pathways offer dimensionally reduced representations of metabolic function, they are modular constructs that do not fully capture cellular function. In MetaCyc (version 25.1), a well-curated metabolic pathway database, only 63.8% of reactions carry pathway annotations, meaning one-third of known enzymatic functions are systematically excluded during pathway-based functional profiling. Therefore, a comprehensive functional profiling requires moving beyond pathway-centric approaches towards multi-resolution functional profiling.

At the core of such multi-resolution profiling lies the gene–protein–reaction (GPR) relationship. Despite the importance of comprehensive GPR mapping for functional characterization, substantial fractions of metagenomic data remain functionally uncharacterized, i.e sequences that cannot be assigned a biochemical function, an annotation gap commonly termed ‘functional dark matter’ [11]. This dark matter arises from two distinct types of causes: genuinely unknown gene functions, where no characterized homologue or protein domain exists, and incomplete GPR mapping and cross-database integration, where the function is known but is never linked to the gene. The latter is methodological, rather than biological, and tractable yet systematically overlooked.

To address this gap, we developed a comprehensive, hierarchical GPR mapping framework that maps UniRef90 gene families (GF) [12] through UniProtKB accession, EC- and protein domain (Pfam [13])-based mapping strategy to reactions across MetaCyc, a well curated database, as well as BioCyc, a more comprehensive database [14]. This modular approach enables functional annotation at multiple resolutions, from individual genes to proteins and enzymatic reactions, allowing researchers to select the appropriate functional granularity and metabolic database for their specific research questions. The framework can be applied directly as custom mapping files with existing functional metagenomics tool, HMP Unified Metabolic Analysis Network (HUMAnN) [6]. We applied our mapping framework to human gut metagenome dataset and compared outcomes with HUMAnN’s default database mapping approach at reaction resolution. Our GPR mapping approach significantly increased detectable metabolic reactions, and their prevalence across samples providing basis for more robust comparative analyses in microbiome studies.

## Methods

### Database sources and versions

**Figure 1.**
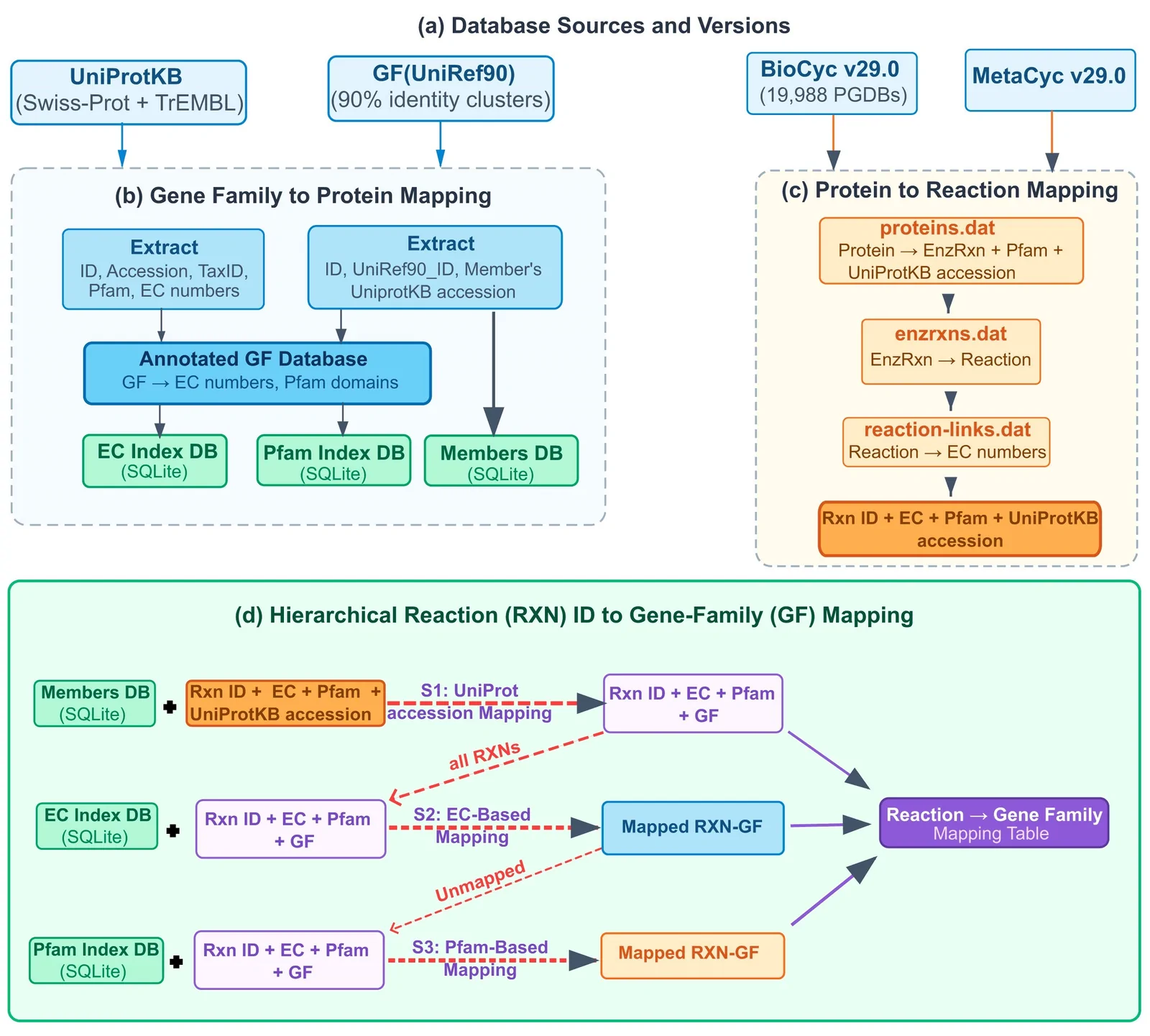
Workflow for constructing the hierarchical reaction-to-GF mapping resource. (**a**) Database sources. Inputs are UniProtKB (Swiss-Prot and TrEMBL) and UniRef90 clusters (90% sequence identity), providing protein-level annotation; and BioCyc v29.0 with MetaCyc v29.0, providing reaction-level information. (**b**) GF-to-protein mapping. UniProtKB entries are parsed for per-protein identifier, accession, taxonomy ID, Pfam domains, and EC numbers, compiled into an annotated GF database and stored as EC Index and Pfam Index SQLite databases. UniRef90 entries are parsed for each cluster identifier and its members’ UniProtKB accessions, stored in a Members SQLite database. (**c**) Protein-to-reaction mapping. BioCyc and MetaCyc flat files are parsed in sequence; proteins.dat (protein to enzymatic reaction, Pfam, UniProtKB accession), enzrxns.dat (enzymatic reaction to reaction), and reaction-links.dat (reaction to EC numbers) to assemble, per reaction, a record of its EC numbers, Pfam domains, and UniProtKB accessions. (**d**) Hierarchical reaction-to-GF mapping. Reaction records from panel (c) are mapped to GF by three sequential strategies, each querying one index from panel (b): S1, UniProt-accession (Members DB); S2, EC number (EC Index DB); and S3, exact Pfam domain-set match (Pfam Index DB). Dashed arrows denote reactions carried between stages (all reactions from S1 to S2; reactions unmapped after S2 to S3); solid arrows denote the per-stage GF assignments, merged into the final mapping table.

We integrated data from three major biological databases to construct comprehensive GPR mappings as shown in Fig. 1. UniProtKB [15] (version 2019_01, including Swiss-Prot and TrEMBL, access date August 27, 2025) and UniRef90 [12] (version 2019_01, clustered at 90% sequence identity, access date August 27, 2025) were obtained from the UniProt Consortium. MetaCyc (version 29.0), a representative, curated, and experimentally elucidated pathway/genome database (PGDB), and BioCyc (version 29.0, access date June 24, 2025), a collection of 20 065 organism/strain-specific PGDBs spanning across all domains of life, were obtained under the appropriate license from SRI International [14].

### Gene family to protein mapping

Protein-level annotations (primarily EC numbers and Pfam domain identifiers) were obtained from UniProtKB by parsing both Swiss-Prot and TrEMBL databases. A unified annotation table was constructed containing unique identifiers (ID), UniProtKB accessions, NCBI taxonomy identifiers, Pfam domain(s), and EC number(s).

In this study, each GF corresponds to a UniRef90 cluster, identified by a cluster ID, e.g. UniRef90_P0AGB6, where P0AGB6 is the UniProtKB accession of the cluster’s representative member. The latter was used to query the annotation table, and the corresponding EC number(s) and Pfam domain identifier(s), when present, were assigned directly to the GF. No annotation aggregation across cluster members was performed. To assess review status, the UniProtKB accession of each UniRef90 cluster representative was compared against the Swiss-Prot accession list, classifying each representative sequence as reviewed if present and unreviewed otherwise.

To support efficient downstream reaction mapping, two SQLite databases were constructed. The first records UniRef90 cluster membership, enabling lookup of representative-to-member relationships. The second stores GF-level annotations in two indexed tables: one for GF–EC number pairs, and one for GF–Pfam domain pairs, the latter including the full sorted Pfam domain composition per GF. Both tables are indexed on EC numbers and Pfam domain composition.

### Protein to reaction mapping

For each PGDB, a multi-step parsing workflow was implemented to progressively link identifiers across flat files. First, protein identifiers were extracted from proteins.dat together with their associated enzymatic-reaction identifiers, Pfam domain identifiers, and UniProtKB accession. Second, enzymatic-reaction identifiers were linked to reaction identifiers via enzrxns.dat. Third, reaction identifiers were parsed from reactions.dat and merged with cross-database reaction links from reaction-links.dat to consolidate EC number annotations per reaction identifier. When multiple proteins, enzymatic-reaction identifiers, or EC numbers were associated with a reaction, all unique associations were retained using a union-based approach. Partial EC numbers were preserved in their original form. Finally, all identifier associations were merged to produce organism-specific reaction annotation files containing reaction identifiers together with their associated EC number(s), Pfam domain(s), and UniProtKB accession(s).

### Hierarchical reaction-to-gene family mapping strategy

We used a hierarchical three-tier mapping strategy to link MetaCyc and BioCyc reaction identifiers to UniRef90 GF, applied independently to each PGDBs.

Step 1: UniProtKB accession-based mapping. Reaction identifiers with associated UniProtKB accession(s) were mapped directly to GF identifiers by querying the cluster membership SQLite database.

Step 2: EC-based mapping. Independently, all reaction identifiers with EC number annotations were mapped to UniRef90 GF identifiers by querying the EC index table. Each EC number was matched as an exact literal string, including partial ECs with unspecified positions (e.g. 1.1.-.-); accordingly, a partial EC was matched only to GF with the identical partial EC string in the index. Where a reaction identifier carries multiple EC numbers, GF assignments obtained across all EC numbers were pooled into a single set per reaction identifier. The GF assignments from Steps 1 and 2 were subsequently pooled for each reaction identifier. As EC numbers directly encode enzymatic function, this step provides high-confidence reaction-to-GF assignments.

Step 3: Pfam-based mapping. For reaction identifiers that could not be assigned to GF following Step 2, Pfam domain identifiers were used as an alternative mapping strategy. Each reaction identifier may carry one or more independent Pfam domain sets, reflecting alternative domain compositions associated with different protein subunits or isoforms. Each independent Pfam domain set was matched separately against the pfam indexed table, where a GF was assigned only when its Pfam domain composition was identical to that of the domain set being matched, that is, both must share exactly the same set of Pfam domain identifiers regardless of their order. GF assignments obtained across all independent Pfam domain sets of a reaction were pooled together with any existing GF assignments carried forward from Step 1, resulting in a single consolidated set of UniRef90 identifiers per reaction. This exact matching strategy captures GF that are well-characterized at the domain level but lack formal EC classification.

The three tiers were combined cumulatively into three mapping strategies: EC, reactions mapped through EC-number matching alone (Step 2); EC+Pfam, the EC set plus reactions additionally mapped through exact Pfam-domain matching (Step 3); and EC+Pfam+UniProtKB accessions, the EC+Pfam set plus reactions recovered through UniProt-accession matching (Step 1) where neither EC nor Pfam annotations exist. Each strategy was applied independently to MetaCyc and BioCyc. For MetaCyc, the strategies were applied directly to a single PGDB. For BioCyc, the strategies were applied independently to each PGDBs; the resulting reaction-to-GF assignments were subsequently merged across all PGDBs within each mapping tier, taking the union of GF assignments per reaction identifier and removing redundant entries. This yields six custom mapping files, each compared against the HUMAnN3 default.

### Functional profiling of shotgun metagenome

Functional profiling of shotgun reads was performed by HUMAnN3 (v3.6.0) [16]. HUMAnN3 internally executes MetaPhlAn (v3.1.0) to infer species-level taxonomic composition based on clade-specific marker genes, using the mpa_v30_CHOCOPhlAn_201901 marker database [16]. Based on the detected taxa, HUMAnN3 constructed a sample-specific pangenome database from ChocoPhlAn and aligned reads to these species-specific reference sequences to identify GF at the nucleotide level. Reads that did not map to the species-specific pangenomes were subsequently translated and searched against the UniRef90 protein database (uniref90_201901b) using DIAMOND to recover additional GF assignments. GF abundances were reported by HUMAnN3 in reads per kilobase (RPK), which normalizes gene length.

For each sample, UniRef90 GF abundance profiles were converted to reaction-level profiles using the humann_regroup_table utility with three mapping strategies (EC, EC+Pfam, EC+Pfam+UniProtKB accessions) applied independently to MetaCyc and BioCyc databases (the ‘Hierarchical reaction-to-gene family mapping strategy’ section), six custom mapping in total, compared to the default HUMAnN3 outputs. At reaction resolution, reads were classified as UNMAPPED (not aligned to GF), UNGROUPED (aligned to GF but not assigned to metabolic reactions), or GROUPED (aligned to GF and assigned to metabolic reactions). To express all three categories as proportions of total reads:


\begin{equation*}\mathrm{UNMAPPED} = \frac{\text{reads not aligned}}{\text{total reads}} \times 100, \quad \mathrm{MAPPED} = 100 - \mathrm{UNMAPPED},\end{equation*}

\begin{equation*}\mathrm{UNGROUPED} = \frac{\mathrm{GF}_{\mathrm{unassigned}}}{\mathrm{GF}_{\mathrm{total}}} \times \mathrm{MAPPED}, \quad \mathrm{GROUPED} = \mathrm{MAPPED} - \mathrm{UNGROUPED},\end{equation*}

where $\mathrm{GF}_{\mathrm{total}}$ is the sum of RPK abundances across all detected GF and $\mathrm{GF}_{\mathrm{unassigned}}$ is the RPK sum of GF with no reaction assignment. As UNMAPPED is determined solely by the alignment step, it is identical across all mappings and presented as a single reference. Differences between mapping strategies are therefore reflected exclusively in the UNGROUPED and GROUPED fractions (arithmetic complements). Paired Wilcoxon signed-rank tests were used to compare each of the approaches against the HUMAnN3 default, with bonferroni correction (*n = 6*; significance threshold *P < .001*). Distributions were visualized using boxplots with swarm plots.

### BioCyc reaction collection and stoichiometric deduplication

Out of 20 065 PGDBs in BioCyc, 77 databases were incomplete and was not considered for analysis. The remaining 19 988 PGDBs were parsed individually, and the reaction identifiers together with their associated stoichiometries were extracted from each database using the parse_pgdb function in cycparser (v1.2.112) (https://gitlab.com/qtb-hhu/cycparser-py). The extracted reactions from each PGDB were assembled into individual SBML models, which were subsequently merged into a single SBML model. When a reaction identifier occurred in multiple PGDBs, only a single copy of the identifier and its associated stoichiometry was retained. The MetaCyc stoichiometry was used whenever available; otherwise, the most frequently observed stoichiometry among the PGDBs containing that reaction identifier was selected. This produced a non-redundant collection of 562 869 reaction identifiers. Stoichiometrically duplicate reactions were identified with the isoform detection function of ScrumPy [17](https://gitlab.com/MarkPoolman/scrumpy), which groups reactions with identical stoichiometry and retains one representative per group.

PGDBs within BioCyc vary substantially in curation depth, ranging from Tier 1 databases such as EcoCyc that undergo intensive manual literature curation to Tier 2/3 PGDBs generated largely by automatically with limited manual review. Because our BioCyc-wide merge combines reaction and protein records across all PGDBs without distinguishing curation tier, BioCyc-derived reaction-to-GF assignments should be interpreted as varying in confidence according to the curation tier of the contributing PGDB(s).

## Results and discussion

### Comprehensive gene–protein–reaction mapping

**Figure 2.**
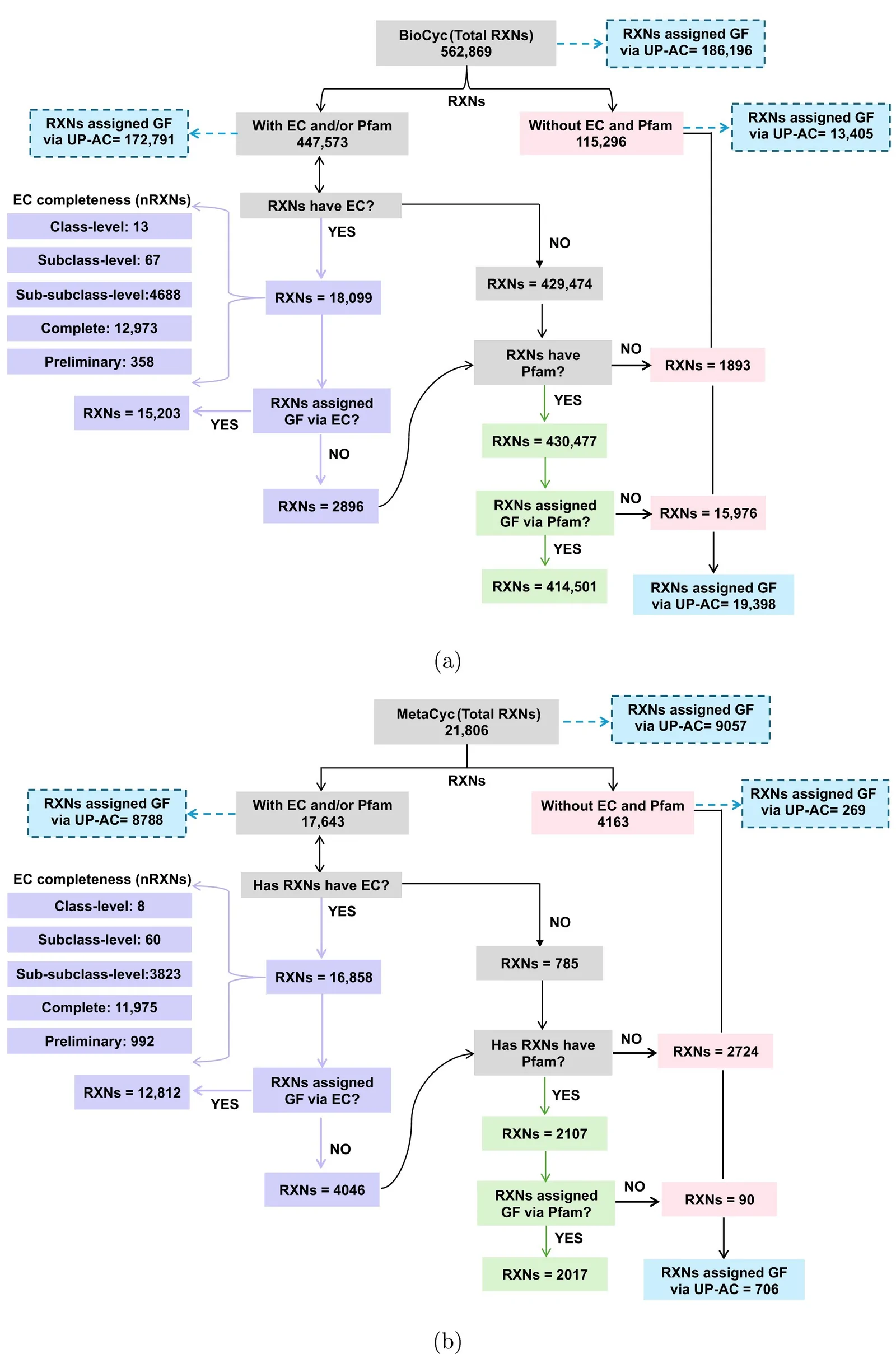
Reaction-level breakdown of GF assignment for (**a**) BioCyc and (**b**) MetaCyc. Each panel partitions the database’s full reaction (RXN) set (562 869 for BioCyc; 21 806 for MetaCyc) and assigns reactions to UniRef90 GF (GF) by three strategies: EC number, Pfam domain, and UniProtKB accession (UP-AC). Reactions are split ‘With EC and/or Pfam’ and ‘Without EC and Pfam’ sets. ‘With EC and/or Pfam’ pass through the EC branch (EC-bearing reactions also stratified by EC completeness) then the Pfam branch, each node showing reaction counts (nRXNs) on its YES/NO paths. UP-AC assignment is given in dashed boxes (whole set and each partition) and a solid box (reactions unassigned after EC and Pfam).

We used a hierarchical three-tier mapping strategy, as described in the ‘Hierarchical reaction-to-gene family mapping strategy’ section and Fig. 1, to systematically map reactions in BioCyc and MetaCyc, to their corresponding UniProtKB accession, EC numbers, and protein domains (Pfam), and subsequently linked these to UniRef90 GF. Figure 2 summarizes the distribution of reactions captured at each mapping level across both BioCyc and MetaCyc databases. Reaction counts refer to unique reaction identifiers unless stated otherwise.

#### Reaction to gene family mapping

Annotation coverage in BioCyc. As shown in Fig. 2a, of the 562 869 reaction identifiers in BioCyc (version 29), ~79.5% (447 573 reaction identifiers) carried EC and-or Pfam based annotations. EC coverage was remarkably limited: only 3.2% (18 099 reaction identifiers) had an EC assignment, and a mere 2.3% (12 973) possessed complete four-digit EC classifications. In contrast, 76.5% (430 477) of BioCyc reaction identifiers are linked to pfam protein domains but lack EC assignments, representing a substantial pool of pfam-supported, domain-characterized reactions that remain inaccessible to EC-only mapping approaches.

Annotation coverage in MetaCyc. As shown in Fig. 2b, MetaCyc exhibits better EC coverage than BioCyc but still shows annotation gaps. Of 21 806 reaction identifiers in MetaCyc, 77.3% carry EC annotations (54.9% have complete four-digit), while 9.6% have pfam assignment but not EC.

From a reaction-to-GF mapping perspective, as shown in Fig. 2a, 2.7% of BioCyc reactions (15 203) mapped to GF via EC numbers alone, providing a high-confidence, EC-supported GPR association. A further 73.6% (414 501 reaction identifiers) were mapped via exact Pfam domain matching, providing lower-confidence domain-supported reaction associations, and an additional 3.4% (19 398) were recovered exclusively through UniProtKB accessions-based mapping where neither EC nor Pfam annotations were available. In total, 20% of reaction identifiers (113 767) could not be mapped to any GF.

In contrast, 58.7% of MetaCyc reactions (12 812) mapped to GF via EC numbers alone, providing a high-confidence, EC-supported GPR association. GPR association for an additional 12.5% reactions could be recovered through Pfam and UniProtKB accessions-based strategy. Despite MetaCyc being manually curated database, 28.7% (6271) of its reactions could not be mapped to GF. Among these reactions, only 839 (3.8%) were annotated as spontaneous reactions not requiring enzymatic catalysis, suggesting that still 25% of reactions have annotation gaps rather than non-enzymatic chemistry.

#### Gene family to reaction mapping

From a GF-to-reaction mapping perspective, 47.8% of the 87 296 736 UniRef90 families lacked both EC and Pfam annotations, likely representing hypothetical proteins or domains not yet characterized. Among annotated GF, 7.61% carried both EC and Pfam assignments, 0.42% had EC only, and 44.2% had Pfam only. Through our hierarchical framework, 31.8% of all UniRef90 families could be mapped to biochemical reactions: 4.15% via EC alone, 7.58% via both EC and Pfam, and 23.8% via Pfam alone. EC-supported assignments represent high-confidence GPR associations, whereas the Pfam-only assignments are lower-confidence, domain-supported candidates that substantially extend coverage beyond EC-based approaches alone but do not guarantee enzymatic function or reaction specificity.

Reviewed Swiss-Prot UniProtKB representation among mapped gene families. The UniRef90 database consists of 87 296 736 clusters. Of these, 0.38% have a reviewed Swiss-Prot UniProtKB representative sequence. For MetaCyc, of the 14 922 360 unique UniRef90 representatives assigned across the 15 535 mapped reaction identifiers, 1.05% have a reviewed Swiss-Prot UniProtKB representative; stratified by tier, 142 512 of 6 244 471 (2.28%) EC-mapped representatives are reviewed versus 43 161 of 10 087 666 (0.43%) Pfam-mapped representatives. For BioCyc, of the 27 932 534 unique UniRef90 representatives assigned across 449 102 mapped reaction identifiers, 0.70% have a reviewed representative, and 160 879 of 8 012 737 (2.01%) EC-mapped representatives are reviewed versus 100 085 of 22 011 737 (0.45%) Pfam-mapped representatives. A total of 84.68% (13 155 out of 15 535) of MetaCyc reactions and, 92.73% (416 446 out of 449 102) BioCyc reactions consist of both reviewed and unreviewed GF, with reviewed representatives consistently enriched among EC-mapped relative to Pfam-mapped reactions.

### Functional profiling of gut microbiome: a case study

**Figure 3.**
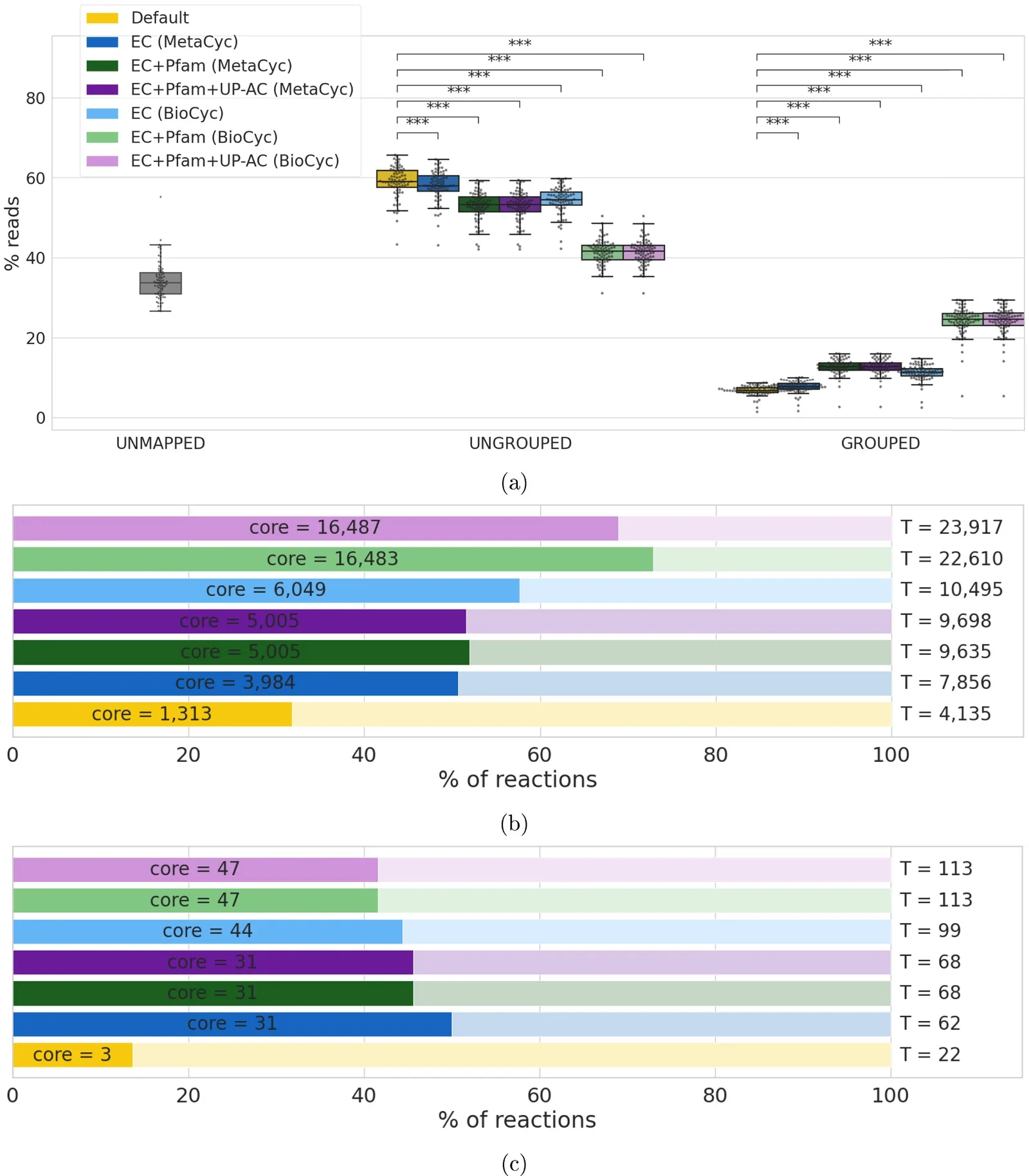
Comprehensive GPR mapping improves functional profiling and reduces data sparsity. The human gut metagenome dataset (BioProject PRJNA749645) was used as the case study. (**a**) Read-fraction distribution across UNMAPPED, UNGROUPED, and GROUPED categories for the HUMAnN3 default and six custom mappings (EC, EC+Pfam, and EC+Pfam+UniProtKB accessions applied to MetaCyc and BioCyc). All six custom mappings significantly reduce the UNGROUPED fraction (paired Wilcoxon signed-rank test, Bonferroni-corrected, *n = 6*; *P < .001*). UNMAPPED: reads not aligned to GF; UNGROUPED: reads aligned to GF but not assigned to reactions; GROUPED: reads assigned to reactions; UP-AC: UniProtKB accessions. (b, c) Reaction coverage and prevalence for each mapping (default and the six custom mappings), plotted as the percentage of reactions (*x*-axis); *T* is the total number of reaction detected in the dataset and core is the number detected consistently across all stratified samples. (**b**) Total and core reactions detected in the dataset; for BioCyc only stoichiometrically unique reactions are reported, whereas default and MetaCyc are reaction-identifier counts. (**c**) Total and core reactions associated with hormone-metabolism; reaction identifier counts is used consistently across all databases.

We applied our GPR mapping framework to human gut metagenomic datasets (BioProject PRJNA749645) to assess how database integration approach affects functional profiles. Functional profiles at reaction resolution were generated using HUMAnN3 pipeline, as described in the ‘Functional profiling of shotgun metagenome’ section. Results in Fig. 3 use percentage-based metrics for cross-database and mapping strategies comparision.

As shown in Fig. 3a, the default HUMAnN3 approach assigns, on average, *≈*6.6% of total reads to metabolic reactions (GROUPED), with *≈*59% of reads aligning to GF that lack reaction assignments (UNGROUPED). EC-only mapping (MetaCyc and BioCyc) yields marginal improvement in GROUPED reads (*≈*7.6% and *≈*11.3%, respectively). Incorporating Pfam domain-based mapping substantially increases reaction-assigned reads: EC+Pfam and EC+Pfam+UniProtKB accession strategies reach *≈*12.7% GROUPED for MetaCyc and *≈*24.2% for BioCyc, corresponding to a 3.7-fold increase over default. All six custom mappings significantly reduced the UNGROUPED fraction relative to default (paired Wilcoxon signed-rank test, Bonferroni-corrected *P < .001*)

As shown in Fig. 3b, the default output identifies 4135 reaction identifiers in the human gut metagenomic dataset. MetaCyc-based custom mappings detect between 7856 and 9698 reaction identifiers (1.9-2.3-fold increase). BioCyc-based mappings detect between 10 521 and 423 635 reaction identifiers; however, this range reflects BioCyc’s inclusion of isoform and organism-specific identifier variants rather than proportionally more unique stoichiometry. Therefore, distinct identifiers with same stoichiometry in BioCyc were collapsed, as described in the ‘BioCyc reaction collection and stoichiometric deduplication’ section, resulting into 24 322 stoichiometrically unique reactions (details of reaction identifiers sharing same stoichiometry is provided in Supplementary Table S1). Ten-thousand five-hundred twenty-one to 423 635 reaction identifiers identified using BioCyc-based mapping corresponds to 10 495–23 917 unique reaction stoichiometry. Transporter reactions, which are less consistently annotated and less precisely defined stoichiometrically, were the main source of this redundancy in BioCyc (Supplementary Table S1).

In addition, reaction prevalence across samples increased substantially, as shown in Fig. 3b. With default HUMAnN3 mapping, 1313 of 4135 detected reaction identifiers (31.75%) were core (i.e present across all stratified samples). EC-based mapping increased core reaction identifiers to 3984 of 7856 (50.71%) for MetaCyc and core stoichiometrically unique reactions to 6049 of 10 495 (57.6%) for BioCyc. Adding Pfam domain-based mapping further increased this to 51.95% for MetaCyc and 73% for BioCyc. This directly addresses the data sparsity challenge that limits statistical power and hinders translational applications [2, 3].

To assess whether the expanded reaction coverage reflects biologically meaningful functional gains, we examined hormone metabolism reactions in BioProject PRJNA749645 dataset [18], where elevated gut microbial steroid hormone biosynthesis (KEGG map00140; 36 EC numbers mapping to 177 MetaCyc reaction identifiers) was linked to therapy resistance in prostate cancer. We assessed coverage and prevalence across 271 reactions from 45 hormone metabolism pathways (comprising KEGG map00140 reactions and additional hormone metabolism pathways identified in MetaCyc; details in Supplementary Table S2), using reaction identifiers across all mapping strategies. At the pathway level, the default HUMAnN3 output does not detect any complete hormone metabolism pathway. It detected 22 of 271 reactions (3 core; 13.6%), whereas custom mapping of MetaCyc detected 62–68 reactions (31 core) (Fig. 3c).

To assess specificity, we examined two targeted reaction sets as positive and negative controls. For the positive control, androgen biosynthesis (six reactions) has been experimentally confirmed in human gut bacteria at the isolate level [18]. The default HUMAnN3 mapping and the MetaCyc-based custom mapping recovered only one of six reactions, whereas BioCyc-based custom mapping recovered four of six. These reactions are present in MetaCyc but were not linked to GF through the MetaCyc mapping rather they were recovered only through BioCyc, whose organism-specific PGDBs carry the strain-level protein associations needed to match them to the gut GF. As a negative control we tested 26 reactions from two vertebrate-specific thyroid-hormone-metabolism pathways (PWY-6260, deiodination; PWY-6261, conjugation/degradation), which should not appear in a human gut metagenome. Neither the default nor any custom mapping strategy detected these reactions, supporting the biological plausibility of the recovered reaction coverage for these tested functions specifically, rather than indicating mapping artefacts. This targeted validation does not establish the global correctness of the broader set of newly assigned reactions though, which would require more extensive experimental or literature-based benchmarking. In addition, we tested for the RuBisCO carboxylase reaction (a plant-specific enzyme), it was detected by BioCyc-based custom mappings but by neither the default nor any MetaCyc-based custom mapping, indicating that this over-prediction stems from database choice rather than the mapping strategy in this case. Together with the androgen-biosynthesis positive control, this result illustrates that BioCyc-derived mappings can both recover reactions missed by MetaCyc and introduce biologically implausible assignments, underscoring the need to interpret reaction assignments with confidence tiers that reflect both the mapping strategy (EC versus Pfam) and the source database (MetaCyc versus BioCyc), rather than treating all expanded mappings as equally reliable.

### Mapping strategies versus databases choice

Our framework is deliberately modular: the three mapping strategies (EC-only, EC+Pfam, and EC+Pfam+UniProtKB accession) are released as separate files for each databases, MetaCyc and BioCyc, giving six mapping files. Rather than imposing a single coverage-specificity trade-off, this lets users match mapping stringency to their research question along two axes, database and mapping strategy. MetaCyc is a highly curated database of experimentally elucidated metabolic pathways and is therefore the more reliable reference. BioCyc extends coverage through organism-specific PGDBs that carry strain-level protein associations but introduces false positive as PGDBs in BioCyc vary in curation depth. Our control analyses (the ‘Functional profiling of gut microbiome: a case study’ section) show both sides of this: BioCyc-based mapping recovers experimentally confirmed androgen reactions missed by MetaCyc, but also detects a plant-specific reaction (RuBisCO), which is likely to be overprediction. Within each database, EC-based mapping is high-confidence and specific, whereas Pfam-based mapping broadens coverage but can misassign reactions when a domain is shared across distinct enzyme families. Therefore, studies requiring high specificity, such as targeted or confirmatory analyses (e.g. attributing a specific transformation to a defined enzyme) or those focused on well-annotated central metabolism, would be best served by the EC and/or MetaCyc-based mapping, whereas exploratory functional surveys and gap-filling step in metabolic network modelling benefit from the broader Pfam and/or BioCyc-based mapping framework.

### Study limitations

The broader Pfam strategies (EC+Pfam and EC+Pfam+UniProtKB accession) increase coverage but trade off specificity (the ‘Mapping strategies vs databases choice’ section). Although we apply the higher-confidence strategies first and only reactions not mapped to GF are propagated through broader Pfam-based approach, specificity cannot be fully verified, because there is no ground-truth reference for the complete metabolic potential of the gut microbiome, or any other microbiome, as it has not been exhaustively cultured or characterized, and many microbial genes still have no experimentally verified function. This is a general constraint for functional metagenomics rather than specific to our approach. Additional limitations to note:

- Because GF annotation is drawn solely from each UniRef90 cluster’s representative sequence, residual within-cluster functional heterogeneity among non-representative members, which are grouped by sequence similarity rather than confirmed functional equivalence, is not captured, and the reaction assignment inferred for a gene family may not hold uniformly for every member of that cluster.
- Although UniRef90 representative selection preferentially retains reviewed Swiss-Prot UniProtKB representative sequences where available, only 0.38% of UniRef90 clusters (334 651 of 87 296 736) have a reviewed representative. Our mapping files do not flag individual mapping records as reviewed Swiss-Prot or not so annotation reliability arising from review status of UniProtKB is not accounted.
- BioCyc-derived mappings inherit the heterogeneous curation quality of their source PGDBs. We do not currently stratify mappings by PGDB curation tier, so BioCyc-based reaction assignments should be treated as variable in confidence rather than uniformly reliable.
- Additionally, BioCyc does not document the evidentiary basis of individual Pfam cross-references, so the provenance of Pfam annotations propagated through our pipeline cannot be verified, and we cannot confirm the Pfam cross-references are exhaustively curated for every protein, so the absence of a Pfam annotation in BioCyc does not necessarily indicate the protein lacks that domain.
- Union-based retention of associations (the ‘Protein to reaction mapping’ section) can produce many-to-many ambiguity between reactions, EC numbers, Pfam domain sets, and GF within a given mapping tier. While GPR relationships can be one-to-one, many-to-one, one-to-many, or many-to-many; the many-to-many associations observed here may therefore reflect genuine metabolic complexity (e.g. isozymes or broad-specificity enzymes), mapping artefacts (e.g. an EC number or Pfam domain shared across functionally unrelated proteins), or both, and distinguishing between these possibilities would require experimental validation beyond the scope of this framework.
- Biological validation (the ‘Functional profiling of gut microbiome: a case study’ section) targeted a small set of hormone-metabolism, positive- and negative-control reactions; it demonstrates biological plausibility for these tested functions but does not establish the global correctness of the full set of newly assigned reactions. Confidence in any individual reaction assignment therefore depends on the mapping tier (EC- versus Pfam-supported) and source database (MetaCyc versus BioCyc) used, as discussed in the ‘Mapping strategies versus databases choice’ section.

### Future directions and broader implications

While our approach substantially increases reads mapped to metabolic functions, a considerable fraction of metagenomic reads still remain unmapped (reads not mapped to genes) or ungrouped (genes not mapped to metabolic function) (Fig. 3a). Presence of these groups is biologically plausible and complete elimination is neither expected nor realistic, due to non-coding regions, sequencing artefacts, and non-metabolic genes. Our framework recovers reactions for the ungrouped fraction and does not change the unmapped fraction, which is fixed by the read-to-GF alignment step. Because it operates only on UniRef90 GF and MetaCyc/BioCyc reactions, the framework is habitat-agnostic and could be valuable in less-characterized environments such as soil or marine microbiomes, where EC-level annotation is sparser than in the well-studied gut and a larger share of reactions is recoverable only through Pfam-based mapping. However, a further systematic investigation is needed to ensure functions are not overlooked due to annotation gaps. While functional metagenomics pipelines remain constrained by cross-database integration, periodic remapping will be essential as curation improves, particularly with AI-based annotation advances.

Pathway-level profiling, while offering a dimensionally reduced summary of metabolic function, introduces systematic bias by collapsing reaction-level evidence into binary or threshold-based presence/absence calls. Reaction-resolution profiling is more insightful than pathway-level resolution, as it grounded in direct GPR associations, but it generates high-dimensional datasets that challenge existing analytical paradigms designed for pathway-level or low-dimensional datasets. Specialized frameworks are needed in the field to handle data dimensionality, tailored to metabolic network topology. As microbiome datasets continue to grow in scale and complexity, such methodological innovations will be essential for extracting biological insights and enhancing translation potential.

Most fundamentally, functional metagenomics demands methodological introspection at the interface of biology, computation, and mathematics, interrogating not just what a tool outputs but how that output is produced. As this work shows, the choice of database, its version, and the mapping thresholds behind a pipeline can shape a functional profile as much as the underlying biology. Unless these choices are scrutinized rather than inherited as defaults, biological interpretation rests on technical and mathematical assumptions that stay invisible to those drawing the biological conclusions.

## Conclusion

Comprehensive functional characterization is essential for translating microbiome research into clinical and biotechnological applications, yet current approaches systematically underestimate metabolic capabilities. This work demonstrates that systematic integration of curated (MetaCyc) and comprehensive (BioCyc) reaction databases with GF through complementary UniProt-accession, EC, and Pfam-based mapping recovers GPR associations for the larger portion of reactions. Applied to a human gut metagenome, these mapping strategies substantially increases the fraction of reads assigned to function, reaction coverage, and the prevalence of reactions across samples. These gains are derived from database integration alone, without predictive algorithms, and demonstrate increased reaction-assignment coverage. The framework identifies a tractable, mapping-related component of functional underannotation in microbiome studies, with confidence in any individual reaction assignment depending on the evidence tier (EC- versus Pfam-supported) and database used.

Cross-database integration is a tractable but systematically overlooked source of this functional underannotation, and addressing it substantially improves functional coverage. Technical fixes alone, however, are not enough. Realising the translational potential of microbiome research also requires a shift in practice: moving beyond pathway-level summaries towards reaction-resolution profiling, choosing the database and mapping stringency that fit the question rather than a fixed default, and scrutinising how functional outputs are produced such as the database dependencies, thresholds, and assumptions built into standard tools.

## Supplementary Material

lqag110_Supplemental_Files

## Data Availability

The step-by-step workflow and scripts used to generate the mapping files, along with the final processed mapping files generated in this study, are archived and accessible via Zenodo: https://doi.org/10.5281/zenodo.20529744 and https://doi.org/10.5281/zenodo.20528513.

## Author declarations

All authors have read and approved the manuscript.
