# Supplementary material for: Identifying fundamental gaps in functional metagenomics: a step towards unlocking microbiome research potential
Source: NAR Genom Bioinform. 2026 Sep 8;8(3):lqag110. doi: 10.1093/nargab/lqag110 (PMC13551040; doi:10.1093/nargab/lqag110)
Supplement: Supplementary file 1 [file lqag110_supplemental_files.zip › Supplementary_Information.pdf]

Supplementary Tables

Supplementary Table 1 (Supplementary_Table_1.txt): Stoichiometric duplicate groups in BioCyc, listing each representative reaction identifier together with the reaction identifiers that share its stoichiometry; Supplementary Table 2 (Supplementary_Table_2.tsv): The 271 hormone-metabolism reactions analysed in the case study, annotated with their MetaCyc pathway identifiers and pathway names.
